# Evaluation of Experimental Nanocomposite-Based Solution and Dentifrice for Reducing Dental Erosive Wear

**DOI:** 10.1590/0103-644020267026

**Published:** 2026-10-19

**Authors:** Cristiane de Melo Alencar, Maria Eduarda Martins Bentes, Karla Lorene de França Leite, Millene de Oliveira Dias, Fernanda Oliveira Miranda Tavares, Gustavo Miranda Rocha, Lucianne Cople Maia

**Affiliations:** 1 Department of Clinic, School of Dentistry, Pará State University Center, PA, Brazil; 2 Department of Pediatric Dentistry and Orthodontics, School of Dentistry, Universidade Federal do Rio de Janeiro, Rio de Janeiro, RJ, Brazil; 3 nstitute of Biophysics Carlos Chagas Filho, Center for Health Sciences, Universidade Federal do Rio de Janeiro, Rio de Janeiro, RJ, Brazil

**Keywords:** Nanotechnology, Tooth Erosion, Mesoporous Silica, Magnesium Hydroxide

## Abstract

To assess the *in vitro* effect of experimental solution and dentifrice on the progression of erosive tooth wear. Bovine enamel (n = 40) and root dentin (n = 40) specimens were pre-eroded (0.3% HCl; pH 1.2; 5 min) and randomized into 4 groups (n = 10, per group): experimental solution based on mesoporous silica containing calcium and fluoride (MSCaNaF); experimental dentifrice based on magnesium and fluoride (MgF_2_) and negative (water, Milli-Q^®^) and positive (stannous-containing dentifrice, Elmex^®^) controls. After half-covering, the specimens were submitted to a second erosive challenge for 5 days (100 μL; 1min; 3×/day). Differences in volumetric roughness (Sa) and tooth structure loss (TSL) by use of 3D noncontact optical profilometry were evaluated. Scanning electron microscopy (SEM) was performed. The data were analyzed by Kruskal-Wallis, Mann-Whitney, and Wilcoxon tests (α=0.05). The intra-group evaluation of Sa showed that all groups differed from the initial condition. For Sa, in relation to enamel and dentin, all experimental groups showed a difference in roughness compared with the negative control (p < 0.05). TSL analysis revealed that the MSCaNaF, MgF_2_, and positive control groups were similar (p>0.05) and more effective compared to the negative control (p<0.05). In the SEM images, the negative control presented the worst loss of dental structure, with more porous enamel. The dentine surface topography pattern showed partially or totally occluded dentinal tubules after treatments, except in the negative control group. The new products based on MSCaNaF and MgF_2_ are effective in controlling the progression of dental erosion, similar to commercial products

## Resumo

Avaliar o efeito *in vitro* de solução e dentifrício experimentais na progressão do desgaste dentário erosivo. Espécimes de esmalte (n = 40) e dentina (n = 40) bovinos foram pré-erodidos (0.3% HCl; pH 1.2; 5 min) e randomizados em quatro grupos (n = 10, por grupo): solução experimental baseada em silica mesoporosa contendo cálcio e flúor (MSCaNaF); dentifrício experimental baseado em magnésio e flúor (MgF_2_) e controles negativo (água, Milli-Q^®^) e positive (dentifrício contend estanho, Elmex^®^). Após metade cobertos, os espécimes foram submetidos a um segundo desafio erosive por 5 dias (100μL; 1min; 3×/dia). Diferenças em rugosidade volumétrica (Sa) e Perda de Estrutura Dentária (PED) foram avaliadas por perfilometria óptica 3D de não-contato. Microscopia Eletrônica de Varredura (MEV) foi realizada. Os dados foram analisados pelos testes de Kruskal-Wallis, Mann Whitney e Wilcoxon (α = 0.05). A avaliação intragrupo de Sa mostrou que todos os grupos diferiram da condição inicial. Para Sa em relação ao esmalte e à dentina, todos os grupos experimentais diferiram do controle negative (p <0.05). A análise da PED revelou que os grupos MSCaNaF, MgF_2_ e controle positive foram similares (p>0.05) e mais efetivos comparados ao controle negative (p<0.05). Nas imagens de MEV, o controle negative apresentou a pior perda de estrutura dentaria, com esmalte mais poroso. O padrão da topografia de superfície da dentina mostrou túbulos dentinários parcial ou totalmente ocluídos após os tratamentos, exceto no grupo do controle negativo. Os novos produtos baseados em MSCaNaF e MgF_2_ são efetivos em controlar a progressão da erosão dentaria, similares ao produto commercial.

**Figure:**
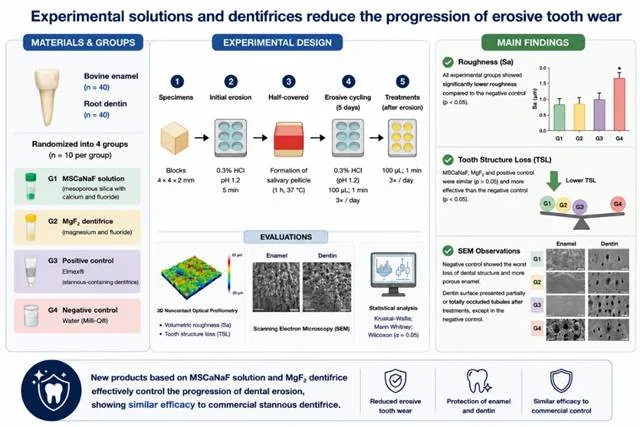

## Introduction

Dental erosion refers to the chemical loss of mineralized tooth substance caused by exposure to acids not derived from oral biofilm, and digestive disorders and dietary factors are the main potential contributing factors ^1^. Exposing enamel to acid causes changes in the microscopical morphology and physical characteristics, such as hardness and surface roughness, and, depending on its severity, can cause dentin exposure ^2^ ^,^ ^3^.

Overall, different products, including solutions, have been assessed to reduce dental erosion. These fluoride products can reduce enamel loss because regular use of fluoride promotes the formation of calcium fluoride, which dissolves less readily than hydroxyapatite ^4^ ^,^ ^5^. In addition, in the literature, the use of novel calcium-based compounds, both in association with or without fluoride, has been proposed to reduce mineral loss ^6^ ^,^ ^7^ ^,^ ^8^. Nanoscale compounds, such as mesoporous silica, can provide chemical and physical improvements in the treatment of enamel or dentin demineralized compared with micrometric compounds, including greater mechanical and thermal stability, higher heat resistance, and lower dissolution ^9^. Furthermore, combining two or more substances into nanoparticles generally yields a product with properties superior to those of the original substances ^10^.

Among the various strategies to control erosive tooth wear, the use of dentifrices containing different active agents may help increase the resistance of the eroded surface to further acids or mechanical impacts ^11^. The literature suggests that erosive tooth wear reduction is greater with SnF2-containing dentifrices than with NaF-containing dentifrices; however, the evidence is insufficient for definitive conclusions ^12^ ^,^ ^13^ ^,^ ^14^. Another dentifrice described is magnesium hydroxide products, bearing in mind their properties, which can represent a promising strategy to counteract erosive tooth wear, as these studies showed that magnesium ions react with enamel during dissolution and precipitation, thereby improving enamel hardness ^15^ ^,^ ^16^.

Nanotechnology has emerged as a promising approach in the remineralization of eroded dental tissues ^17^ ^,^ ^18^ ^,^ ^19^ ^,^ ^20^ ^,^ ^21^. The application of nanomaterials, such as nano-hydroxyapatite, has been shown to effectively restore enamel and dentin by facilitating the deposition of minerals at the nanoscale, closely mimicking the natural composition of tooth structures. Studies have demonstrated that these nanoparticles can penetrate subsurface lesions, promoting the formation of a new mineralized layer that enhances the mechanical properties and resistance of the tooth to further demineralization ^22^ ^,^ ^23^. 

The aim of the present study was to assess the *in vitro* effects of two new products, a solution of mesoporous silica with calcium and fluoride (MSCaNaF) and a dentifrice of magnesium hydroxide and fluoride (MgF_2_), on the progression of erosive tooth wear in enamel and root dentin. The null hypothesis of this study was that the application of these products does not reduce volumetric roughness (Sa) or tooth structure loss (TSL) after erosive challenge compared with negative and positive controls.

## Materials and methods

### Preparation of the experimental agents

Mesoporous silica (MS) based nanocomposites were obtained by the nanoprecipitation technique, varying the molar ratio of water/tetraethoxysilane (TEOS), NH_3_/TEOS, and the amount of cetyltrimethylammonium bromide. All characterization analysis was described according to previously reported in the literature by Leite et al. ^(^ ^24^, sodium fluoride (1.36% NaF, 6135 ppm of F-) solutions (Aldrich Chemical Co^®^, Saint Louis, USA) were added with calcium (Ca) to produce the experimental solution nanocomposite: MSCaNaF.

Experimental dentifrice was prepared using magnesium hydroxide (Mg(OH)_2_), added with sodium fluoride (NaF, 1450 ppm; pH = 9.91), and other basic ingredients: calcium carbonate, magnesium hydroxide, sodium lauryl sulfate, and magnesium sulfate. This agent was synthesized according to Medvecky et al*.* ^25^

Dentifrice commercial containing 1400 ppm F− as AmF, 700 ppm F− as NaF, 3500 ppm Sn2^+^; pH 4.5 (Elmex^®^ Erosion Protection Toothpaste; Germany) as positive control, and the distilled and deionized water (Milli-Q Merck^®^, Saint Louis, USA) was used as negative control. 

The experimental dentifrice (MgF_2_) and stannous-containing dentifrice (Elmex^®^) are available as toothpastes, so a 1:3 slurry was prepared by combining toothpaste (one-third) with distilled and deionized water (two-thirds; Milli-Q^®^).

### Study design and simple size

This *in vitro* study, a randomized, controlled, single-masked study, used a sample size (n=10 per group and substrate) based on a previous study ^8^ that used similar compounds, in which the TSL values for the negative control and MSCa were 37.96±18.88 μm and 12.03±8.28 μm, respectively. These values were assumed for the sample size calculation, assuming 0.8 statistical power to detect a 50% significant difference between groups, using a one-tailed test with a 5% significance level (BioEstat 5.3^®^, Instituto de Desenvolvimento Sustentável Mamirauá, Tefé, Brazil). A calculated sample size of n=8 per treatment group was required to complete the study. With an estimated 25% loss, at least n=10 specimens per substrate should be selected.

### Specimen preparation

Enamel specimens (4×4×4 mm) were prepared from bovine crowns as described previously ^26^. Surface hardness was determined on enamel ^27^. Enamel blocks within ±10% of the baseline microhardness mean (319.23 kgF/mm^2^) were selected and randomized into groups. From these groups, 40 sound enamel blocks were selected. 

Root dentin specimens (4×4×4 mm) were prepared from the cervical third of the bovine incisor tooth root. The root dentin blocks were cut and polished in a similar way to that described for the enamel blocks. Afterward, these specimens were cleaned and dried for evaluation of the surface by two researchers independently, and they were checked to determine whether they had a smooth surface and no fractures or other visual defects. If they showed a dentin defect, the specimens were excluded from the study; if they did not have a smooth surface, they were polished again. From these groups, 40 root dentin blocks were selected. Finally, the prepared blocks were stored in a humid environment (Milli-Q^®^ water) at 4°C. 

### Initial erosion and protection of specimens

After random distribution of the specimens (Microsoft Excel^®^) into each group (n=10 per group and substrate), all specimens were transferred to a 12-well polystyrene plate (model K12-024, Kasvi^®^, São José do Pinhais, Brazil). Previously treated all the specimens were subjected to artificial saliva at 37 °C for 2 h (concentration of components: 0.96 g/1000 mL - KCl; NaCl; MgCl_2_; K_2_HPO_4_; CaCl_2_; Carboxymethyl cellulose; Sorbitol 70%; Nipagin; Nipazole, and deionized water). After removal of the saliva, an initial erosion lesion was created by applying 0.3% HCl (5 ml per specimen), pH 1.2, for 5 minutes; after this time, the surface was washed with distilled water for 30s. 

After that, half of the surface of the specimens was covered with acid-resistant nail varnish to create an unexposed area (the eroded initial area) and an exposed area. Also, the root dentin specimens were protected on their surfaces with non-plasticized polyvinyl chloride tape, leaving a 4 × 2 mm^2^ windows uncovered, which was exposed to the treatments and demineralization/ remineralization (DES-RE) cycles using artificial saliva ^3^.

### Treatment with materials and acid cycling

After protecting the eroded samples, the treatments were applied by an independent researcher who was not involved in the other stages of the study to minimize operator-related bias. The researcher was unaware of the group allocation; however, complete blinding was not feasible due to inherent differences in the materials' consistency and visual appearance. The test products were applied to the treatment area using a pipette (100 μL) and allowed to remain on the surface for 10 minutes per treatment. The treatments were performed according to the following groups: MSCaNaF, MgF2, Elmex®, and Milli-Q® water. This study evaluated the effect of a single application of these products.

The second erosive challenge was performed by immersing the blocks in a low-pH solution (0.3% HCl, pH 1.2) to simulate an erosive condition and in an artificial saliva (pH 7.0) to simulate clinical practice. All specimens were subjected to a 3-day series of erosion cycles, performed with fresh solutions 3 times daily for 5 minutes each. After the erosion protocol, the specimens were rinsed in Milli-Q^®^ water for five seconds and transferred to artificial saliva for two hours. After the last day of the erosive challenge, the specimens were also stored in artificial saliva overnight. The solutions were changed daily to avoid oversaturation, and the experiment was carried out at 37 ºC.

### Data collection and analysis

Following the removal of the protective coating covering half of the surfaces of all specimens (the untreated areas), the topography of the blocks (including both treated and untreated areas) was analyzed. No baseline measurement was performed, as a split-sample design was used, in which each specimen served as its own control, allowing direct comparison between treated and untreated areas.

The roughness of the surface specimens was measured by 3D noncontact profilometry (Nanovea PS50 Optical, Nanovea, Irvine, USA). The one mm^2^ assessment area on the blocks was standardized. An image-capture chromatic confocal sensor using a white-light axial source, with a scan velocity of 2 mm/s and a refractive index of 10,000, was used. The Sa (ISO 25178) (250 μm^2^) value was calculated as Sa = Sa1 − Sa2, where Sa1 was the average roughness in the untreated area and Sa2 was the average roughness in the treated area, per block. TSL was defined as the difference in height between the untreated and treated areas per block. During the calculation, three linear measurements were completed in triplicate. The examiner of the study was blind to the analyses. The same examiner had access to the blocks only via random numbers previously demarcated ^3^.

A qualitative analysis of surface topography was performed using Scanning Electron Microscopy (SEM). Specimens (n=2 per group) were gold-sputtered prior to SEM visualization. SEM (Fei Quanta 250®, Thermo Fisher Scientific®, Waltham, USA) was operated at 20 kV. Photomicrographs were obtained at 5.000× magnification. The photomicrographs obtained showed the microstructural characteristics of the treated and untreated areas ^24^.

### Statistical analysis

The normality of the data was tested for all variables using the Shapiro-Wilk test. The Kruskal-Wallis, Mann-Whitney, and Wilcoxon tests (p < 0.05) were used to profile the parameters. The photomicrographs of the surface obtained via noncontact profilometry and the SEM findings were analyzed descriptively, including an evaluation of changes in the sample surface between the untreated (before treatment) and treated (after treatment) areas after erosive challenge.

## Results

There was a significant increase in Sa between the initially eroded and the post-erosive challenge in the negative control group (p < 0.05). On the other hand, there was a significant reduction in MSCaNaF and MgF2 in the positive group (p < 0.05) compared to the initially eroded group. Therefore, the intra-group evaluation of Sa showed that all groups differed from the initial condition. Still considering the values of Sa, in relation to enamel and root dentin, all experimental groups showed a difference in roughness with the negative control (p <0.05) (Figures 1 and 2).

The TSL analysis revealed that the MSCaNaF, MgF_2_, and positive groups were similar (p >0.05) and more effective at minimizing tooth loss than the negative control (p <0.05) in both substrates (Figure 3).

Application of the products altered the surface topography of the samples across all groups; the rough appearance of the samples was more pronounced in the negative control group. Qualitative analysis of photomicrographs of the surface confirmed the superiority of the treatments relative to the negative control (Figure 4).

**Figure 1:**
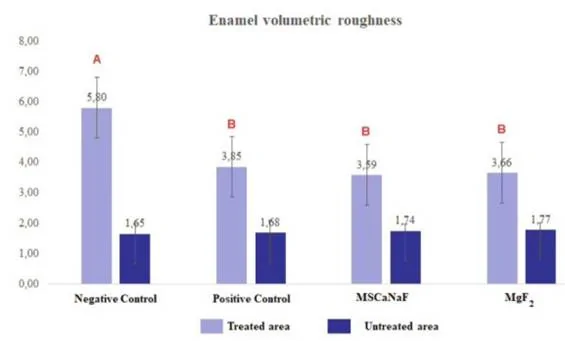
Values of Sa for the enamel specimens obtained via noncontact profilometry after erosion challenge. Uppercase letters show differences between the groups (p < 0.05).

**Figure 2:**
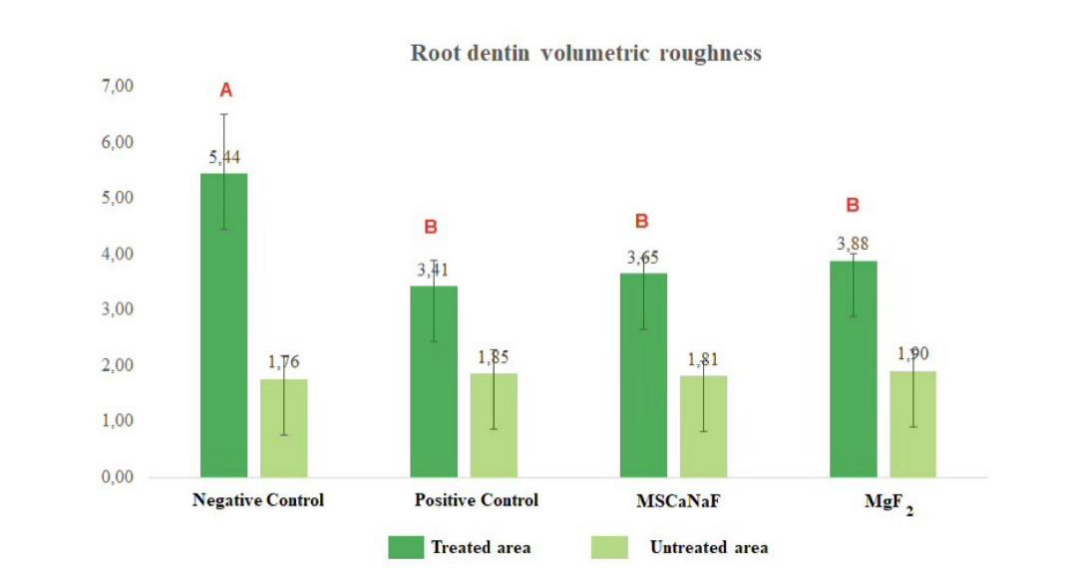
Values of Sa for the root dentin specimens obtained via noncontact profilometry after erosion challenge. Uppercase letters show differences between the groups (p < 0.05).

**Figure 3:**
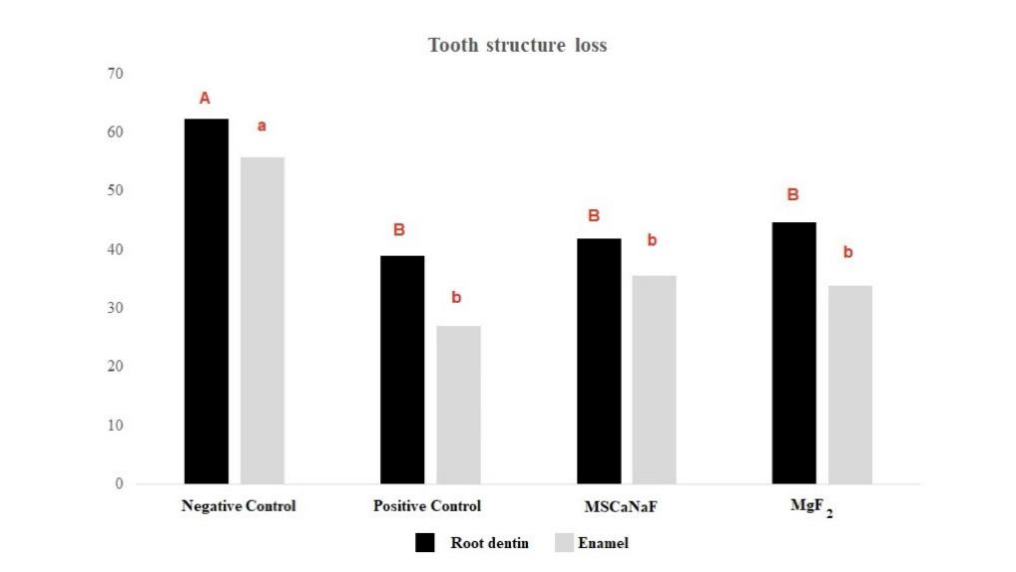
Values of TSL for the specimens obtained via noncontact profilometry after erosion challenge. Uppercase letters show differences between the groups for the root dentin specimens (p < 0.05), and lowercase letters represent differences for the enamel specimens (p < 0.05).

In the SEM images, the negative control presented the worst loss of dental structure, with more porous enamel. This group also showed more porous enamel compared with the other groups, indicating a greater loss of tooth structure in the exposed area following the erosion process. Ultimately, the MSCaNaF, MgF_2_, and positive group showed more effective protection against erosion, with lower porosity and less mineral loss. Also, the dentine surface topography pattern was compared between groups (intergroup comparison) to determine the presence of partially or fully occluded dentinal tubules after treatment. Material deposits were observed over the dentin tubules for all treatment groups, except in the dentin tubules of the control group (Figure 5). 

**Figure 4:**
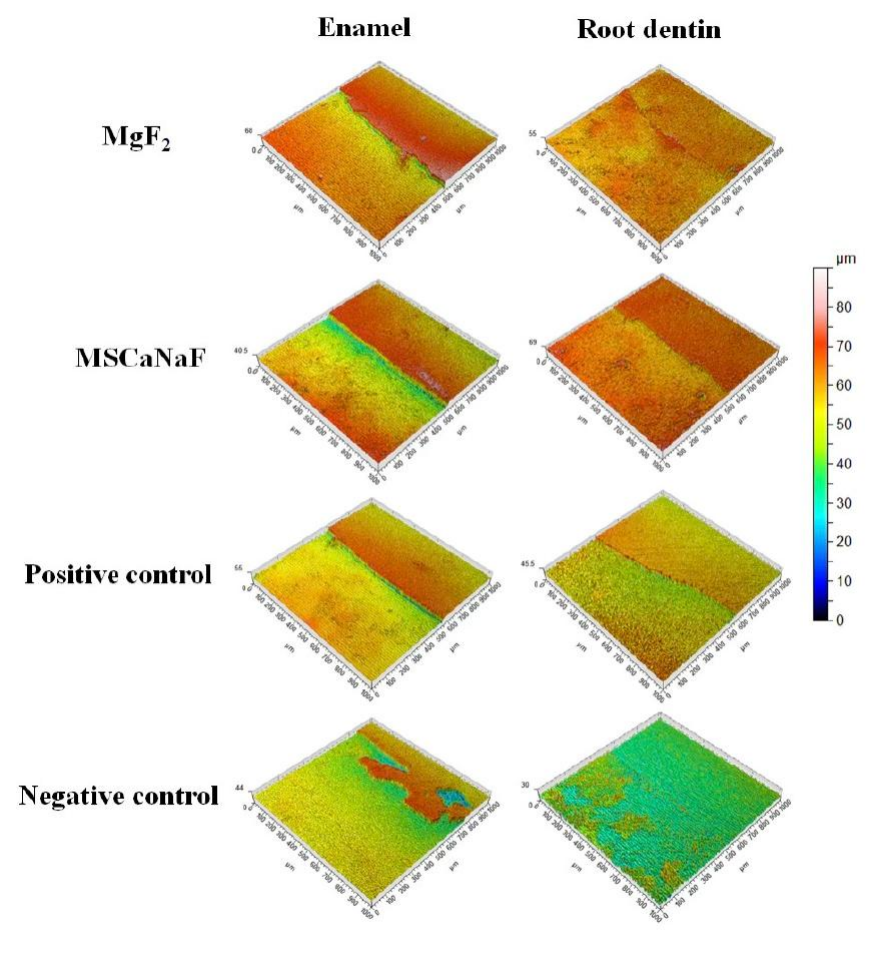
Photomicrographs of the surface of the specimens after the erosive challenge obtained via noncontact profilometry. The left side of the image represents the Sa eroded.

**Figure 5:**
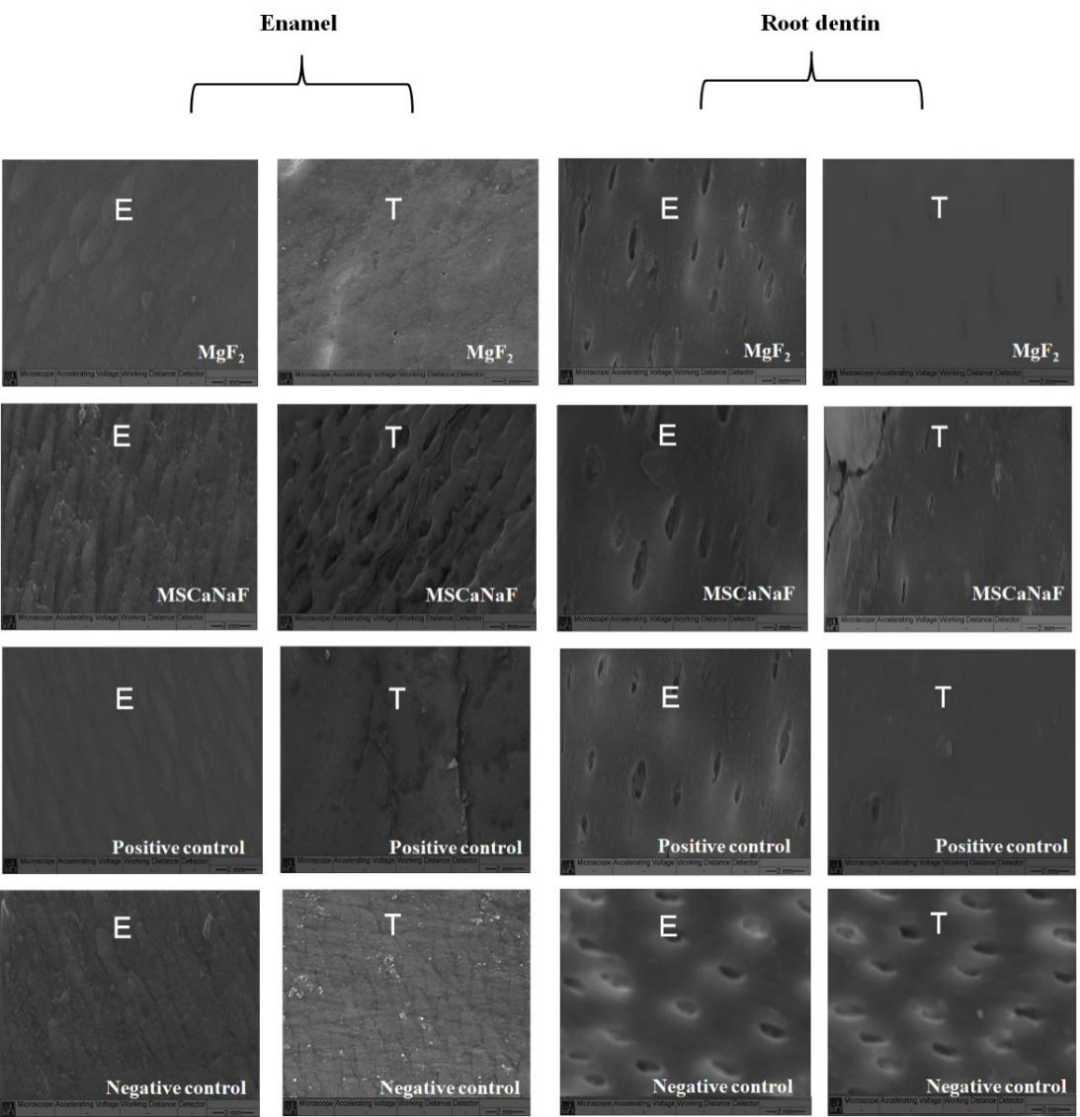
Surface of specimens after the erosive challenge assessed by scanning electron microscopy at 5,000x. E = eroded and T=treated.

## Discussion

The findings of this *in vitro* study suggest that mesoporous silica nanoparticles (MS-nanoparticles) combined with fluoride and calcium are promising alternatives for managing erosive tooth wear. These nanoparticles, widely recognized for their large surface area and high pore volume, have shown significant potential in drug delivery and biomedicine applications, as demonstrated by Gu et al. ^28^ and Yan et al. ^29^. Their adaptability and efficiency highlight the need for further exploration of such innovative materials in dental practice. Moreover, the study reinforces the importance of magnesium hydroxide and sodium fluoride-containing dentifrices in mitigating enamel and root dentin erosion caused by dietary acids. These findings emphasize the relevance of incorporating advanced nanotechnology-based solutions into strategies for erosion prevention and treatment. 

The null hypothesis that there would be no difference in the effect of the tested solutions and dentifrices on erosive tooth wear caused by acidic challenges was rejected. This was based on the significant differences observed in Sa and TSL values between treated and untreated conditions, as well as the superior performance of MSCaNaF, MgF_2_, and the positive control group in reducing surface roughness and tooth structure loss compared to the negative control.

Several studies report recent advances in MS-nanoparticles, in which this product is more effective at preventing tooth enamel loss than the positive control group ^6^ ^,^ ^7^ ^,^ ^8^ ^,^ ^30^ ^,^ ^31^. In dentistry, only three studies have evaluated MSCaNaF-based materials ^24^ ^,^ ^30^ ^,^ ^31^ ^,^ ^32^ for the remineralization of dental tissues. However, only one of these studies investigated their anti-erosive potential. Moreover, no prior findings were identified concerning the effects of these novel mesoporous silica nanoparticles combined with fluoride products on enamel and root dentin.

Mesoporous silica nanoparticles have emerged as promising agents in combating dental erosion due to their unique structural properties and functional capabilities. Their high surface area and porous architecture enable efficient loading and controlled release of therapeutic agents, such as calcium and fluoride ions, which are essential for remineralizing demineralized dental tissues. Upon application, MSNs facilitate the deposition of these ions onto enamel and dentin surfaces, promoting the formation of a new mineralized layer that enhances the mechanical properties and the tooth's resistance to further demineralization. This process effectively restores the integrity of eroded dental tissues, as demonstrated in studies evaluating the efficacy of calcium mesoporous silica nanoparticles in reducing tooth structure loss ^33^. 

Additionally, MSNs can be functionalized to deliver bioactive compounds that inhibit matrix metalloproteinases (MMPs), enzymes responsible for degrading the organic matrix in dentin during erosion. For instance, quercetin-encapsulated hollow mesoporous silica nanocomposites have been shown to effectively occlude dentinal tubules and preserve the demineralized organic matrix, thereby reducing dentine erosion and abrasion ^30^. 

In this study, the experimental magnesium hydroxide dentifrice was as effective as the commercial product in protecting dental substrates against acid erosion. This result may be explained by the acid buffering that occurs immediately upon contact with Mg(OH)_2_, which produces a salt (Mg_2_C_6_H_4_O_7_) and H_2_O via a neutralization reaction. Furthermore, the abrasive (calcium carbonate) present in the dentifrice can react with magnesium hydroxide and form byproducts of reaction (MgCO_3_ and Ca(OH)_2_), making residual acid neutralization possible ^15^ ^,^ ^16^. A systematic review of topical agents for nonrestorative management of dental erosion found that incorporating magnesium compounds into oral care products could offer additional benefits for erosion prevention. The review emphasized the need for more clinical studies to confirm these findings and to establish standardized protocols for the use of Mg(OH)₂ in dental practice ^34^.

The results of the present study indicate that MgF₂ dentifrices may offer protection for enamel and dentin against mild erosion. However, this conclusion is limited by the absence of abrasion testing in the experimental design. Previous studies have reported that the protective effect of MgF₂ dentifrices is diminished when erosion occurs in conjunction with mechanical abrasion ^15^. Despite this limitation, the erosion model employed in the present study demonstrated that all experimental products, including MgF₂, performed comparably to the stannous-containing dentifrice. This suggests that fluoride-based formulations are effective in mitigating the progression of erosive tooth wear under the tested conditions.

Regarding the morphological characteristics of enamel and root dentin, the groups treated with MSCaNaF and MgF_2_, and the positive group, showed a decrease in surface roughness. This effect may be attributed to the presence of sodium fluoride (NaF) in the various treatments, which likely acts as a protective physical barrier. By inhibiting direct contact of acid with the tooth surface, NaF reduces demineralization. Additionally, it serves as a fluoride reservoir, facilitating the precipitation of a calcium fluoride-like layer (CaF₂) ^4^, which further enhances the tooth's resistance to erosive challenges. Although the experimental groups do not have equal fluoride concentrations, these products can exhibit gradual fluoride release due to their nanometric characteristics. 

The morphological features of the enamel were supported by surface photomicrographs, which revealed increased exposure of enamel prisms in the negative control group. In contrast, the root dentin showed greater dentinal tubule obliteration in the treated groups than in the negative control, indicating enhanced protective effects of the treatments. These results may be related not only to sodium fluoride but also to its association with the salivary film.

Saliva plays a crucial role in preventing dental erosion through several mechanisms. It acts as a natural buffer, neutralizing acids in the oral cavity, and facilitates the clearance of erosive agents. Additionally, saliva is supersaturated with minerals such as calcium and phosphate, which are essential for the remineralization of tooth enamel ^35^. The salivary pellicle, a protein-rich film that forms on the tooth surface, serves as a protective barrier against acid attacks. The incorporation of sodium fluoride into this salivary film enhances its protective properties. Fluoride ions promote the formation of fluorapatite, a more acid-resistant mineral phase, thereby increasing enamel resistance to demineralization. Studies have demonstrated that the application of sodium fluoride can significantly reduce enamel erosion by reinforcing the salivary pellicle and facilitating remineralization processes ^36^.

Further clinical studies are necessary to evaluate the effects of these experimental products on dental erosion under various formulations and concentrations. These studies should also assess their efficacy over extended durations, and with repeated, periodic exposure to better understand their long-term protective potential.

## Conclusion

MSCaNaF and MgF_2_ were able to control the progression of enamel and root dentin erosion, as they were similar to a stannous-containing dentifrice and superior to the negative control on all parameters analyzed.

## Data Availability

The research data are available upon request

## References

[1] Yip K, Lam PPY, Yiu CKY (2022). Prevalence and associated factors of erosive tooth wear among preschool children: A systematic review and meta-analysis. Healthcare (Basel).

[2] Al-Amri I, Albounni R, Binalrimal S (2021). Evaluation of the effect of soft drinks on the surface roughness of dental enamel in natural human teeth. F1000Res.

[3] Alencar CM, Leite KLF, Ortiz MIG, Magno MB, Rocha GM, Silva CM (2020). Morphological and chemical effects of in-office and at-home desensitising agents containing sodium fluoride on eroded root dentin. Arch Oral Biol.

[4] Bezerra SJC, Trevisan LR, Viana IEL, Lopes RM, Pereira DL, Aranha ACC (2019). Er,Cr:YSGG laser associated with acidulated phosphate fluoride gel (1.23% F) for prevention and control of dentin erosion progression. Lasers Med Sci.

[5] Chevitarese AB, França Leite KL, Marañón-Vásquez GA, Masterson D, Pithon M, Maia LC (2022). Effectiveness of titanium tetrafluoride to prevent or treat dental caries and tooth erosion: A systematic review. Acta Odontol Scand.

[6] Justino IBS, Alexandria AK, Canto FMT, Leite KLF, Vieira TI, Cabral LM (2020). Comparative effect of calcium mesoporous silica versus calcium and/or fluoride products on the reduction of erosive tooth wear and abrasive enamel lesion. Pesqui Bras Odontopediatria Clin Integr.

[7] Canto FMT, Alexandria AK, Justino IBDS, Rocha GM, Cabral LM, Ferreira RDS (2020). The use of a new calcium mesoporous silica nanoparticle versus calcium and/or fluoride products in reducing the progression of dental erosion. J Appl Oral Sci.

[8] Canto FMT, Alexandria AK, Vieira TI, Justino IBDS, Cabral LM, Silva RFD (2020). Comparative effect of calcium mesoporous silica versus calcium and/or fluoride products against dental erosion. Braz Dent J.

[9] Huang YR, Wu IT, Chen CC, Ding SJ (2020). In vitro comparisons of microscale and nanoscale calcium silicate particles. J Mater Chem B.

[10] Komasa S, Okazaki J (2022). Advances in dental bio-nanomaterials. Materials (Basel).

[11] Magalhães AC, Wiegand A, Buzalaf MAR (2014). Use of dentifrices to prevent erosive tooth wear: Harmful or helpful?. Braz Oral Res.

[12] Trentin GA, Mendes LT, Silva BSD, Casagrande L, Araújo FB, Lenzi TL (2021). Reduction in erosive tooth wear using stannous fluoride-containing dentifrices: A meta-analysis. Braz Oral Res.

[13] Fiorillo L, Cervino G, Herford AS, Laino L, Cicciù M (2020). Stannous fluoride effects on enamel: A systematic review. Biomimetics (Basel).

[14] Zanatta RF, Caneppele TMF, Scaramucci T, El Dib R, Maia LC, Ferreira DMTP (2020). Protective effect of fluorides on erosion and erosion/abrasion in enamel: A systematic review and meta-analysis of randomized in situ trials. Arch Oral Biol.

[15] Passos VF, Rodrigues Gerage LK, Lima Santiago S (2017). Magnesium hydroxide-based dentifrice as an anti-erosive agent in an in situ intrinsic erosion model. Am J Dent.

[16] Passos VF, Rodrigues LKA, Santiago SL (2018). The effect of magnesium hydroxide-containing dentifrice using an extrinsic and intrinsic erosion cycling model. Arch Oral Biol.

[17] De Lavôr JR, Fernandes NLS, De Sousa EBG, Da Cunha JL, Meira IA, Correia Sampaio F (2021). Nanoencapsulated fluoride as a remineralization option for dental erosion: An in vitro study. Acta Odontol Scand.

[18] Fernando JR, Shen P, Sim CPC, Chen YY, Walker GD, Yuan Y (2019). Self-assembly of dental surface nanofilaments and remineralisation by SnF2 and CPP-ACP nanocomplexes. Sci Rep.

[19] Onwubu SC, Mdluli PS, Singh S, Nyembe S, Thakur R (2018). An in situ evaluation of the protective effect of nano eggshell/titanium dioxide against erosive acids. Int J Dent.

[20] Chawhuaveang DD, Yu OY, Yin IX, Lam WYH, Chu CH (2022). Topical agents for nonrestorative management of dental erosion: A narrative review. Healthcare (Basel).

[21] Kwek SX, Mian M, Hall C, Xie Z, Yong R, Kaidonis J (2015). Nanoscratch testing for the assessment of enamel demineralization under conditions simulating wine erosion. Aust Dent J.

[22] Alencar CM, De Paula BLF, Guanipa Ortiz MI, Magno MB, Silva CM, Maia LC (2019). Clinical efficacy of nano-hydroxyapatite in dentin hypersensitivity: A systematic review and meta-analysis. J Dent.

[23] Ortiz MIG, De Oliveira SG, Alencar CM, Aguiar FHB, Lima DANL (2024). Remineralizing effect of the association of nano-hydroxyapatite and fluoride in the treatment of initial lesions of the enamel: A systematic review. J Dent.

[24] Leite KLF, Vieira TI, Alexandria AK, Silva RFD, Silva ASS, Lopes RT (2021). In vitro effect of experimental nanocomposites solutions on the prevention of dental caries around orthodontic brackets. Braz Dent J.

[25] Medvecky L, Stulajterova R, Giretova M, Mincik J, Vojtko M, Balko J (2018). Effect of tetracalcium phosphate/monetite toothpaste on dentin remineralization and tubule occlusion in vitro. Dent Mater.

[26] Hara AT, Queiroz CS, Paes Leme AF, Serra MC, Cury JA (2003). Caries progression and inhibition in human and bovine root dentine in situ. Caries Res.

[27] Cury JA, Rebelo MAB, Del Bel Cury AA, Derbyshire MTVC, Taubchoury CPM (2000). Biochemical composition and cariogenicity of dental plaque formed in the presence of sucrose of glucose and fructose. Caries Res.

[28] Gu J, Huang M, Liu J, Li Y, Zhao W, Shi J (2012). Calcium-doped mesoporous silica nanoparticles as efficient alendronate delivery vehicles. New J Chem.

[29] Yan H, Yang H, Li K, Yu J, Huang C (2017). Effects of chlorhexidine-encapsulated mesoporous silica nanoparticles on the anti-biofilm and mechanical properties of glass ionomer cement. Molecules.

[30] Chen JM, Cheng YL, Yang MH, Su C, Yu H (2024). Enhancing the inhibition of dental erosion and abrasion with quercetin-encapsulated hollow mesoporous silica nanocomposites. Front Bioeng Biotechnol.

[31] Canto FMT, Alexandria AK, Justino IBDS, Rocha GM, Cabral LM, Ferreira RDS (2020). The use of a new calcium mesoporous silica nanoparticle versus calcium and/or fluoride products in reducing the progression of dental erosion. J Appl Oral Sci.

[32] Leite KLF, Martins ML, Dias MO, Tavares FOM, Justino IBDS, Cabral LM (2023). In vitro and in situ caries-preventive effect of a new combined fluoride and calcium experimental nanocomposite solution. Braz Dent J.

[33] Canto FMT, Alexandria AK, Vieira TI, Justino IBDS, Cabral LM, Silva RFD (2020). Comparative effect of calcium mesoporous silica versus calcium and/or fluoride products against dental erosion. Braz Dent J.

[34] Chawhuaveang DD, Yu OY, Yin IX, Lam WYH, Chu CH (2022). Topical agents for nonrestorative management of dental erosion: A narrative review. Healthcare (Basel).

[35] Buzalaf MAR, Hannas AR, Kato MT (2012). Saliva and dental erosion. J Appl Oral Sci.

[36] Chawhuaveang DD, Lam WYH, Chu CH, Yu OY (2024). Preventing dentin erosion with silver diamine fluoride and salivary pellicle: An in vitro study. Clin Oral Invest.

